# A Tabular View of the Treatment of Uterine Hemorrhages

**Published:** 1843-06

**Authors:** William Camps

**Affiliations:** M. D., Edin.


*A tabular view of the treatment of Uterine Hæmorrhages.*—By William Camps, M. D., Edin.

### HÆMORRHAGE BEFORE PARTURITION.

A. *Slight Hæmorrhage.*—Horizontal position; perfect repose; cool air; cool acidulous fluids; low diet; bleeding, if there be symptoms of plethora. The bladder and rectum to be emptied.

B. *Severe Hæmorrhage.*—The same treatment as in A., except the bleeding (1). At first, cold applications; then the ergot of rye, in doses of 12 grains, repeated three times, at intervals of ten minutes. And if these means are insufficient to arrest the hæmorrhage, plug the vagina, or in some especial cases rupture the membranes (2).

### HÆMORRHAGE DURING PARTURITION.

*Slight Hæmorrhage.*

Os uteri not dilated, and not dilatable.

Membranes entire:—The same means as in A., except the bleeding, which is not indicated unless the symptoms of plethora are exceedingly pronounced.

Membranes ruptured:—As above.

Os uteri dilated.

Membranes entire:—The same means as in A.; then wait, or rupture the membranes (3).

Membranes ruptured:—The same means as in A., and wait; if the pains are feeble or slow, give the ergot of rye (4).

*Severe Hæmorrhage.*

Os uteri not dilated, and not dilatable.

Membranes entire:—The same treatment as in A., except the bleeding; then the refrigerants, as cold applications. In case these fail, and if the pains are feeble, the ergot of rye; then rupture the membranes. Lastly, if the state of the os uteri will not allow of turning, plug the vagina.

Membranes ruptured:—The same treatment as in A.; refrigerants; ergot of rye if the pains be slow or feeble. In case these fail, compression of the uterus; plug the vagina; introduce the hand, and deliver by turning (5).

Os uteri dilated, or dilatable.

Membranes entire:—Rupture the membranes (6); and if this does not succeed, introduce the hand and turn, or apply the forceps.

Membranes ruptured:—Turning, if the head of the child has not descended into the cavity of the pelvis (7). Apply the forceps if the head of the child have already descended into the cavity of the pelvis. Simple extraction if the child present by breech, knees, or feet.

(1). *The ergot of rye* is employed here as hæmostatic; in the case supposed, there is not, at present, uterine pains; it is not probable that the employment of the ergot of rye should produce them, for hitherto this remedy appears to possess the property of increasing the uterine contractions only when they occur spontaneously, and not that of exciting them, when they do not already exist.

(2). *The plug* will, in the first place, arrest the hæmorrhage; then, by the retention of the blood, and by its presence, it will irritate the *neck* and *orifice* of the uterus, and it will induce the expulsive contractions. These will dilate the *os uteri*, and this dilatation will allow either the rupture of the membranes, or the termination of the accouchment.

(3). This rupture can have no inconvenience; it is a means of preventing the increase of the hæmorrhage. We may always dispense with it, and rest satisfied with waiting until the progress of the labor shall have arrested the hæmorrhage:

the last method is after all perhaps the most prudent. A little *more* or a little *less* tendency to the increase of the hæmorrhage should determine the choice of the one or of the other method.

1st. *Wait,* if the hæmorrhage do not increase in any degree, and still more so if it diminish.

2d. *Rupture the membranes* if there be any tendency to increase of the hæmorrhage; this rupture will be profitably preceded or followed by the administration of some doses of the ergot of rye, if the uterine pains are feeble or slow.

(4). It may be asked, if it would not be proper to terminate the accouchement in this case, since the parts concerned seem disposed to this termination. We think that if the fœtus presents in the usual manner, it is better not to be officious with the application of the *forceps* or *turning*, because the employment of these means would be more severe than the *slight* hæmorrhage which appears to demand their use.

(5). This case is one of great delicacy; the application of the plug here requires great caution; for when the vagina is closed up, the blood may possibly accumulate in the cavity of the uterus, so that the patient may be lost, although no blood make its appearance externally; and the danger will be so much the greater, as the uterus shall have been more developed before the rupture of the membranes, and as the uterine contractions shall be more feeble. Thc application of the plug should be preferred, to the termination of the accouchement, only when the uterine contractions are sufficiently strong, and when, at the rupture of the membranes, only a very small quantity of the liquor amnii shall escape from the uterus. Again, the application of the plug demands great care of and attention to the patient, and should be followed by the application of a bandage round the abdomen sufficiently tight to resist the enlargement of the uterus. On the contrary, when the uterine contractions are feeble, when a large quantity of the liquor amnii shall have escaped at the moment of the rupture of the membranes, it will be necessary to overcome the resistance offered by the state of the os uteri, and terminate the accouchment by *turning*.

(6). In this case we may be surprised at the advice to *rupture the membranes,* and *wait,* before adopting any other method, according as the retraction of the uterus may have, or may not have, arrested the hæmorrhage; it seems so important, both to the mother and to the infant, that the birth of the latter should be the result of the uterine contractions alone, rather than of manual interference, very often difficult, that it is very desirable to take the chance of a spontaneous accouchment at all times when there is the probability of obtaining it. It is to be understood that this recommendation to *wait*, is only admissible in case the uterine contractions are neither feeble nor slow.

(7). We may certainly in this case have recourse to the *forceps*, but the employment of this instrument, when the head of the child is above the orifice, and not engaged in the cavity of the pelvis, frequently offers sufficient difficulty to render the delivery by turning preferable.

It will be seen that the indications are based on the slightness or severity of the hæmorrhage, and not on the circumstance of the insertion or non-insertion of the placenta on the neck of the uterus; not that this circumstance is a matter of indifference, for almost always the hæmorrhage produced by the detachment of the placenta inserted over the orifice of the uterus, is one of a severe and serious character, and demands immediately the employment of the means indicated for severe hæmorrhages. Sometimes the insertion of the placenta on the neck of the uterus occasions only a slight hæmorrhage: the author does not consider, then, as do the greater part of accoucheurs, that the insertion of the placenta on the neck of the uterus requires, in every case, the speedy termination of the accouchment, yet it may modify the employment of the means above indicated. For instance, if in a case of severe hæmorrhage the placenta covers entirely the os uteri, we cannot have recourse to the simple rupture of the membranes, as we could if such were not the case. If the os uteri be neither sufficiently *dilated*, or sufficiently *dilatable*, to allow the introduction of the hand, it will be necessary to employ the plug; if, on the contrary, it be sufficiently *dilated*, or sufficiently *dilatable*, it will be necessary to detach one of the sides of the placenta, in order to make a passage into the cavity of the uterus, and deliver by turning; but if a portion only of the placenta be inserted on the os uteri, leaving exposed a part of the membranes, we may proceed as if the placenta were not inserted at the os uteri. In no case does it seem advisable to make a passage through the placenta, as some accoucheurs have recommended. Lastly, if the placenta, pushed by the head or the breech of the fœtus, be entirely, or almost entirely detached, and has passed beyond the orifice of the uterus, we must extract it before the fœtus, for this organ is useless in these circumstances, and its presence in the vagina is an obstacle to the free exercise of the hand, or of instruments.—*Med. Ex. and Retros. of the Med. Sci.*, from *Lond. Med. Gaz.* Jan. 13, 1843.

